# Psychometric properties of the Kidney Disease Quality of Life short form 36 (KDQOL-36) scale for the assessment of quality of life in Colombian patients with chronic kidney disease on dialysis

**DOI:** 10.1007/s11255-024-03940-x

**Published:** 2024-02-20

**Authors:** Martha Carolina Valderrama-Rios, Ricardo Sánchez, Mauricio Sanabria

**Affiliations:** 1 Clinical Research Institute, School of Medicine, Universidad Nacional de Colombia, Bogotá, DC Colombia; 2 Baxter Renal Care Services-Latin America, Bogotá, DC Colombia

**Keywords:** Kidney Failure, Chronic, Quality of Life, Validation Studies, Psychometrics, Colombia (MeSH)

## Abstract

**Purpose:**

Considering the importance of incorporating quality of life (QoL) construct during the health care of patients with stage 5 chronic kidney disease (CKD) on dialysis, it is necessary to have evidence on the clinimetric properties of the instruments used for its measurement. This study aimed to establish the clinimetric properties of the Kidney Disease Quality of Life Short Form 36 (KDQOL-36) scale in patients with stage 5 CKD on dialysis in Colombia.

**Methods:**

A scale validation study was conducted using the classical test theory methodology. The statistical analysis included exploratory factor analysis (EFA) and confirmatory (CFA) techniques performed on two independent subsamples; concurrent criterion validity assessments; internal consistency using four different coefficients; test–retest reliability; and sensitivity to change using mixed model for repeated measures.

**Results:**

The KDQOL-36 scale was applied to 506 patients with a diagnosis of stage 5 CKD on dialysis, attended in five renal units in Colombia. The EFA endorsed the three-factor structure of the scale, and the CFA showed an adequate fit of both the original and empirical models. Spearman's correlation coefficient values ≥0.50 were found between the domains of the CKD-specific core of the KDQOL-36 scale and the KDQ. Cronbach's alpha, McDonald's omega, Greatest lower bound (GLB), and Guttman's lambda coefficients were ≥0.89, indicating a high degree of consistency. A high level of concordance correlation was found between the two moments of application of the instrument, with values for Lin's concordance correlation coefficient ≥0.7. The application of the instrument after experiencing an event that could modify the quality of life showed statistically significant differences in the scores obtained.

**Conclusion:**

The KDQOL-36 scale is an adequate instrument for measuring QoL in Colombian patients with stage 5 CKD on dialysis.

**Supplementary Information:**

The online version contains supplementary material available at 10.1007/s11255-024-03940-x.

## Background

Chronic kidney disease (CKD) in stage 5 also called kidney failure requires kidney replacement therapy (KRT) as part of the treatment, through peritoneal dialysis (PD), hemodialysis (HD), or kidney transplant [1, 2]. During the last decade, a steady increase in the prevalence of KRT for patients with stage 5 CKD has been documented in different countries worldwide, with HD being the most widely used KRT [3–5].

Patients on dialysis bear a significant burden of symptoms, experienced as part of the natural course of the disease, or concerning medication, dialysis, or dietary and lifestyle modifications, which are necessary as part of the treatment [6], with an impact on the quality of life (QOL) construct [7], and clearly on health-related quality of life (HRQoL) [8, 9]. The effect of CKD on QoL has been described in numerous studies, with some of the consistently reported findings being, (I) the decreased QoL in patients with CKD compared to the general population; (II) the progressive and significant worsening of QoL in relation to the progression of CKD; and (III) the improved QoL in kidney transplant recipients compared to patients on dialysis [10–15]. The role of the HRQoL construct as a significant predictor of morbidity and mortality in patients with CKD has also been widely documented [16–20].

The assessment of constructs, such as QoL, which, by their nature, cannot be assessed by conventional diagnostic tests, requires the use of patient-reported outcomes (PROs) consolidated into valid and reliable instruments called patient-reported outcome measures (PROMs) [21]. For the measurement of aspects related to the health status of CKD patients, numerous generic and disease-specific PROMs are currently available [22, 23]. The quality of the results obtained through a PROM will depend on the measurement properties with which the instrument quantifies the construct of interest in the target population [24]. That is why to use an instrument, it is necessary to carry out the processes of translation and cultural adaptation [25] if it has been originally developed in another population and to have evidence on the measurement properties of the instrument in the population of interest [24]. The Kidney Disease Quality of Life short form (KDQOL-SF) of 80 items and the Kidney Disease Quality of Life short form 36 (KDQOL-36) of 36 item [26, 27] are instruments with the best evidence of adequate clinimetric properties for measuring QoL in CKD patients [28, 29]. This has prompted the translation, cross-cultural adaptation, and validation for its use in different populations around the world [30–36].

Although the cross-cultural adaptation of the Spanish version of the KDQOL-36 carried out in Colombian patients is available [37], there is no validation of the instrument in the Colombian population to date. Considering the importance of assessing HRQoL in CKD patients on KRT, and the lack of an adequately validated instrument for doing so in Colombia, therefore, this study aimed to establish the clinimetric properties of the KDQOL-36 instrument in Colombian patients with stage 5 CKD on dialysis.

## Methods

A scale validation study was conducted from the perspective of classical test theory (CTT).

***Participants***: Adult patients with a diagnosis of stage 5 CKD on dialysis attended in five renal units of the Baxter Renal Care Services® network in Bogota, Colombia. Patients were recruited by non-probabilistic, sequential, and convenience sampling, applying the following inclusion criteria: (I) being 18 years of age or older; (II) being Spanish speaking; and (III) having been in Colombia for the last 10 years. Patients with cognitive or sensory alterations that prevented the adequate application of the instrument were excluded. Sample size calculation was conducted for each of the components of the scale validation process. For the analysis of the validity of the proposed content using polychoric correlation methods, a sample of no less than 250 patients is suggested [38–40], so a total sample of 500 patients was considered, 250 in PD and 250 in HD. For the analyses of concurrent criterion validity, internal consistency, and test–retest reliability, the sample size calculations were performed using the PASS® statistical program, assuming a significance level of 5% and a power of 80%. For the concurrent criterion validity analysis, the estimated sample size was 70 patients, considering a population correlation coefficient of 0.4 for the null hypothesis (H0) and 0.5 for the alternative hypothesis (Ha) [41, 42]. For the internal consistency analysis, a sample size of 101 patients was estimated, taking Cronbach's alpha correlation coefficient values of 0.7 for the H0 and 0.8 for the Ha [43, 44]. For the reliability analysis using the test–retest method, assuming Lin’s concordance correlation coefficient (CCC) of 0.8 for the H0 and 0.9 for the Ha [45], the estimated sample size was 100 patients. For the proposed sensitivity to change analysis using a mixed model for repeated measures (MMRM), the sample size was calculated using the GLIMMPSE® program, taking into account the non-independence of three repeated measures over time and the distribution of patients in two dialysis modalities; considering a difference of at least 2 points and 0.5 points in the standard deviation in the score obtained between the different moments of measurement [46], the estimated sample size was 351 patients [47].

***Instrument***: The KDQOL-36 is a 5-point Likert scale with a generic core and a CKD-specific component. The generic core is measured by the 12-item Short Form Health Survey (SF-12), which consists of 12 items conducted to the physical and mental components, whose scores are converted into mean scores of 50 and standard deviations of 10, whereby values above 50 indicate a better health status than the reference population [48]. The reliability assessment and estimation of normative values of the SF-12 among Colombian adults are available for this country [49]. The CKD-specific component has 24 kidney disease targeted items, distributed in three domains: burden of kidney disease (4 items), symptoms and problems of kidney disease (12 items), and effects of kidney disease (8 items). Item 28, which is part of the symptoms and problems domain, has two wording options depending on the dialysis modality: 28a Hemodialysis patient only “Problems with your access site?” or 28b Peritoneal dialysis patient only “problems with your catheter site?”. The pre-coded numerical values for each item are linearly transformed to a range from 0 to 100, such that for each domain higher scores indicate a better level of HRQoL [27].

***Statistical analysis***: The sociodemographic and clinical data of the participants were analyzed by descriptive statistics, using percentages for categorical variables; and means or medians, with the respective standard deviation (SD) or interquartile range (IQR), for continuous variables.

***Content validity***: It was estimated by sequentially employing the statistical techniques of exploratory factor analysis (EFA) and confirmatory factor analysis (CFA), in two independent subsamples [50, 51]. For the EFA, Bartlett's test of sphericity and the Kaiser Meyer-Olkin test (KMO) were used to check the suitability of the correlation matrix for factor analysis [52, 53]. The number of factors to be analyzed was determined using the Kaiser criterion, the percentage of total variance explained, the eigenvalue sedimentation plot analysis, and parallel analysis [54–59]. To define the factor structure, factor loadings ≥0.3 were considered [60]. To the initial orthogonal solution, orthogonal (varimax) and then oblique (promax and oblimin) rotations were performed in order to select the solution with the best clinically interpretable model. For the CFA, considering the ordinal nature of the scale items, the estimation of the models was performed from a polychoric correlation matrix, using the weighted least squares (WLS) method [61, 62]. To assess the goodness of fit of the models, measures of absolute fit and incremental fit [63] were used, with the following specified values indicating adequate fit [64–66]: chi-square/degrees of freedom (X^2^/df; values <3), root mean square error approximation index (RMSEA; values <0.08), standardized root mean square error (SRMR; values <0.08), and values >0.9 for Comparative Fit Index (CFI), Incremental Fit Index (IFI), Tucker-Lewis Index (TLI) or Non-normalized Fit Index (NNFI), and Goodness-of-Fit Index (GFI).

***Concurrent criterion validity***: Through the concurrent application with another scale that measures the same construct. Considering that in Colombia there is no validated instrument for the evaluation of HRQoL in patients with stage 5 CKD on dialysis, it was considered necessary to perform the translation and cross-cultural adaptation for the Colombian population of the Kidney Disease Questionnaire (KDQ), an instrument also designed to measure the construct of QoL in patients with CKD on KRT, which consists of 26 items distributed in five domains: Physical symptoms (6 items), fatigue (6 items), depression (5 items), frustration (3 items), and relationships with others (6 items), the original version of which is in English [67, 68]. The translation and cross-cultural adaptation processes were performed following the recommendations suggested by the EORTC (European Organization for Research and Treatment of Cancer) Quality of Life Group [25]. Once the two instruments were applied, the scores for each of the domains of both instruments were calculated, the Shapiro–Wilk statistical test was given to determine whether the data had a normal distribution, and then, the Spearman correlation coefficients were calculated between the scores of the CKD-specific domains of the KDQOL-36 and the scores of the domains of the KDQ.

***Internal consistency***: It was performed by estimating four of the suggested coefficients: Cronbach's alpha, McDonald's omega, greatest lower bound (GLB), and Guttman's lambda [69–71], calculated for the CKD-specific core of the scale, for each of the three domains, and through an analysis with item removal.

***Reliability***: Using the test–retest method, the instrument was applied at a second time 8–10 days after the first test, during which time the HRQoL construct remained stable. Lin’s CCC was used, and the dispersion of the correlation and concordance was evaluated graphically using Bland and Altman's goodness-of-fit plots [45, 72].

***Sensitivity to change***: We compared the scores obtained in each of the domains of the CKD-specific component of the KDQOL-36 at three different moments of application of the instrument: (I) baseline; (II) when experiencing an event that could modify the HRQoL; and (III) once the event had ended, again considering the stability of the construct. For this purpose, MMRM were used, taking into account the presence of fixed and random effects, given by the non-independence of the three moments of application of the instrument and also by the distribution of patients in two dialysis modalities, with both between-subjects and within-subjects effects.

The CFA and the calculation of Cronbach's alpha, GLB, and Guttman's lambda coefficients were performed using the R programming language, through RStudio version 1.4.1106 using the libraries lavaan, psych, paran, polycor, and semPlot [73–77]. The remaining analyses of the validation and descriptive statistical component were performed with the STATA 17® program.

## Results

***Characteristics of the participants***: The total sample included 506 patients with stage 5 CKD on dialysis, 50% on HD, and 50% on PD. In the total sample, 61% of the patients were male; the median age was 57.73 years (IQR = 43.75–67.21); the characteristics of the participants in the total sample and according to dialysis modality are shown in Table 1.

**Table 1:** Sociodemographic and clinical characteristics of the study population

	Total sample *n* = 506	Hemodialysis *n* = 253	Peritoneal dialysis *n* = 253
**Gender**, ***n*** **(%)**			
Male	309 (61)	165 (65)	144 (57)
Female	197 (39)	88 (35)	109 (43)
**Age, median (IQR)**	57.73 (23.45)	58.24 (22.09)	57.18 (25.09)
**Educational status**, ***n*** **(%)**			
Read and write	7 (1)	6 (2)	1 (0.5)
Primary	164 (32)	87 (34)	77 (30)
Secondary	266 (53)	128 (51)	138 (55)
College	48 (9)	20 (8)	28 (11)
Postgraduate	13 (3)	5 (2)	8 (3)
None	8 (2)	7 (3)	1 (0.5)
**Occupation**, ***n*** **(%)**			
Unemployed	14 (3)	11 (4)	3 (1)
Employed	95 (19)	37 (15)	58 (23)
Freelancer	59 (11)	30 (12)	29 (11)
At home	121 (24)	73 (29)	48 (19)
Student	20 (4)	6 (2)	14 (6)
Retired	49 (10)	32 (13)	17 (7)
Other	110 (22)	46 (18)	64 (25)
Unknown	38 (7)	18 (7)	20 (8)
**Marital status**, ***n*** **(%)**			
Single	157 (31)	72 (29)	85 (33)
Married	189 (37)	109 (43)	80 (32)
Common-law	100 (20)	44 (17)	56 (22)
Divorced	24 (5)	11 (4)	13 (5)
Widowed	26 (5)	14 (6)	12 (5)
Unknown	10 (2)	3 (1)	7 (3)
**Socioeconomic stratum**^**a**^, ***n*** **(%)**			
1 lower-low	31 (6)	15 (6)	16 (6)
2 low	200 (40)	95 (37)	105 (42)
3 upper-low	234 (46)	126 (50)	108 (43)
4 medium	37 (7.4)	14 (5.5)	23 (9)
5 medium–high	2 (0.4)	2 (1)	0
6 high	1 (0.2)	1 (0.5)	0
**Cause of CKD**, ***n*** **(%)**			
Arterial Hypertension	142 (28)	80 (32)	62 (25)
Diabetes Mellitus	140 (28)	79 (31)	62 (24)
Autoimmune Glomerulonephritis	82 (16)	32 (13)	50 (20)
Obstructive	30 (6)	14 (5.5)	16 (6)
Polycystic kidney disease	19 (4)	9 (4)	10 (4)
Chronic Tubulointerstitial Nephritis	6 (1)	2 (0.5)	4 (1)
Other	39 (8)	16 (6)	23 (9)
Unknown	48 (9)	21 (8)	27 (11)
**Time in KRT in years, median (IQR)**	2.9 (4.29)	3.5 (5.26)	2.5 (3.19)

^a^Stratum 1 corresponds to users with lower economic resources, beneficiaries of public utility subsidies, while stratum 6 corresponds to users with higher economic resources, who pay a surcharge (contribution) on the value of public utilities

***Description of CKD-specific component of KDQOL-36 scores***: The lowest scores, suggesting a greater compromise or decrease in quality of life, were observed in the burden of kidney disease domain with a median of 43.75 (IQR = 25–75), followed by the effects of kidney disease domain with a median of 75 (IQR = 53.12–87.5), and finally the symptoms and problems of kidney disease domain with a median of 81.25 (IQR = 68.75–91.66). Scores in each domain by dialysis modality are shown in the Supplementary material, Figure S1.

***EFA***: The total sample of 506 patients was divided by simple random sampling into two, “subsample 1” and “subsample 2,” each consisting of 253 patients including both dialysis modalities. EFA was performed in the first sample and CFA in the second sample. The results of Bartlett's test of sphericity (χ2 (276) = 2010.685; *p* = 0.000) and the KMO test (0.894) allowed us to conclude that the correlation matrix was suitable for factor analyses. Considering that, in the initial orthogonal solution, the first three factors were found to explain 93% of the variance and had eigenvalues greater than 1.0, the characteristics of the Cattell’s scree plot, and the parallel analysis with the principal factor method, the three-factor analysis was considered adequate. The factorial solution with the best clinical interpretability was the oblique rotation (promax) (Table 2). Factor one with five items that include aspects related to the perception of interference or burden of kidney disease in life; factor two with 11 items, that gather aspects related to the physical symptoms of the disease; and factor three with seven items, that include aspects related to the limitations or effects of kidney disease in daily life. Item 28 “problems with your access site?” or “problems with your catheter site?” showed the highest uniqueness value (0.96) and did not obtain an adequate factor load in any of the three domains.

**Table 2:** Factor structure of the CKD-specific core in the KDQOL-36 scale, oblique rotation (promax)

Item	Factor 1	Factor 2	Factor 3	Uniqueness
i13. My kidney disease interferes too much with my life	0.65			0.45
i14. Too much of my time is spent dealing with my kidney disease	0.62			0.51
i15. I feel frustrated dealing with my kidney disease	0.65			0.52
i16. I feel like a burden on my family	0.62			0.60
i31. Your ability to work around the house?	0.30			0.50
i17. Soreness in your muscles?		0.48		0.61
i18. Chest pain?		0.46		0.76
i19. Cramps		0.34		0.86
i20. Itchy skin?		0.41		0.75
i21. Dry skin?		0.38		0.73
i22. Shortness of breath?		0.57		0.60
i23. Faintness or dizziness?		0.54		0.66
i24. Lack of appetite?		0.55		0.67
i25. Washed out or drained?		0.63		0.37
i26. Numbness in hands or feet?		0.64		0.60
i27. Nausea or upset stomach?		0.54		0.66
i29. Fluid restriction?			0.67	0.52
i30. Dietary restriction?			0.70	0.54
i32. Your ability to travel?			0.46	0.61
i33. Being dependent on doctors and other medical staff?			0.58	0.58
i34. Stress or worries caused by kidney disease?			0.38	0.51
i35. Your sex life?			0.33	0.72
i36. Your personal appearance?			0.55	0.48
i28a. (Hemodialysis patient only) ¿Problems with your access site?	0.09	0.10	0.03	0.96
i28b. (Peritoneal dialysis patient only) ¿Problems with your catheter site?				

**Fig. 1:**
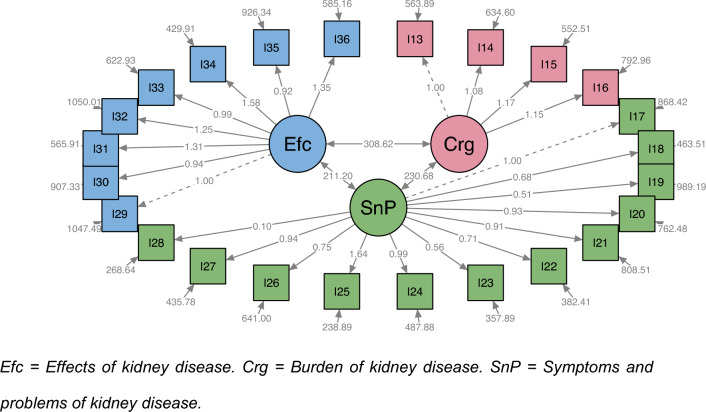
System of structural equations for the CKD-specific core of the KDQOL-36, original model

**Fig. 2:**
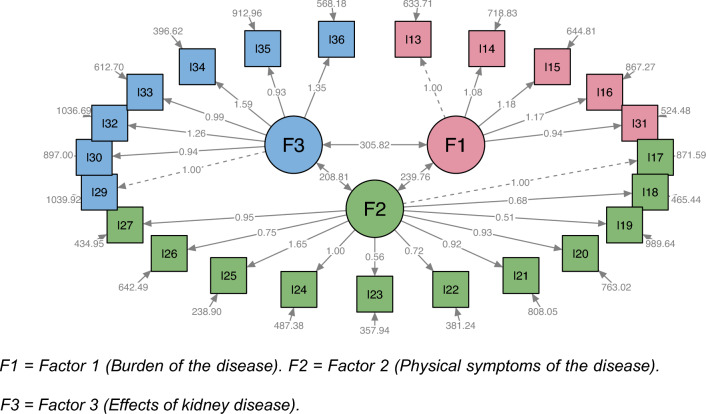
System of structural equations for the CKD-specific core of the KDQOL-36, empirical model

***CFA****:* For this component, using subsample 2, the original model reported by the author [25] and the empirical model resulting from the EFA were evaluated. Figures 1 and 2 show the system of structural equations for both factor structures; the ovals represent the latent variables (domains), the squares represent the observed variables (items), the arrows in a single direction indicate the domain-item causal relationships, the arrows in two directions indicate the correlations between domains, and the arrows in dashed lines correspond to loadings that are set with a value of 1 to estimate the coefficients of the models. The goodness-of-fit estimators obtained for each of the models are presented in Table 3, with values for each of the estimators that indicate an adequate fit of both models and are very similar between them.

**Table 3:** Goodness-of-fit estimators for the evaluated models

Model	X^2^/dF^a^	RMSEA^b^	SRMR^c^	CFI^d^	IFI^e^	TLI^f^	GFI^g^
Original	1.06	0.017	0.06	0.99	0.99	0.99	0.97
Empirical	1.14	0.025	0.06	0.99	0.99	0.99	0.97

^a^Ratio of chi-square/degrees of freedom

^b^Root Mean Square Error Approximation Index

^c^Standardized Root Mean Square Error

^d^Comparative Fit Index

^e^Incremental Fit Index

^f^Tucker-Lewis’s Index

^g^Goodness-of-Fit Index

***Concurrent criterion validity***: Instruments KDQOL-36 and KDQ were applied to 199 patients, 100 patients on HD, and 99 patients on PD. Across the total sample, for each of the three domains of the CKD-specific component of the KDQOL-36, all correlations were statistically different from zero, with values obtained from Spearman's correlation coefficient that were overall >0.50. The highest correlations (ranging from 0.62 to 0.75) were found in the KDQOL-36 burden of kidney disease domain, with the KDQ scores for depression, relationships with others, and frustration; and in the KDQOL-36 symptoms and problems of kidney disease domain, with the KDQ scores for physical symptoms and fatigue; correlations that are clinically plausible and do have an adequate interpretation. Coefficients according to dialysis modality are shown in Table 4.

**Table 4:** Spearman's correlation coefficients for the CKD-specific core of the KDQOL-36 scale

	KDQOL-36 D1	KDQOL-36 D2	KDQOL-36 D3
**Total sample** *n* = 199*			
KDQ D1	0.38	0.66	0.55
KDQ D2	0.62	0.62	0.56
KDQ D3	0.75	0.48	0.64
KDQ D4	0.73	0.51	0.61
KDQ D5	0.72	0.42	0.62
**Hemodialysis** ***n*** = 100*			
KDQ D1	0.33	0.65	0.49
KDQ D2	0.62	0.70	0.51
KDQ D3	0.73	0.58	0.60
KDQ D4	0.75	0.54	0.53
KDQ D5^h^	0.69	0.50	0.62
**Peritoneal dialysis** ***n*** = 99*			
KDQ D1	0.42	0.68	0.60
KDQ D2	0.62	0.57	0.62
KDQ D3	0.77	0.37	0.69
KDQ D4	0.71	0.50	0.70
KDQ D5	0.75	0.35	0.63

*All correlation coefficients with *p* values < 0.01. KDQOL-36 D1 = Burden of kidney disease. KDQOL-36 D2 = Symptoms and problems of kidney disease. KDQOL-36 D3 = Effects of kidney disease. KDQ D1 = Physical symptoms. KDQ D2 = Fatigue. KDQ D3 = Depression. KDQ D4 = Relationship. KDQ D5 = Frustration

***Internal consistency***: The analysis of the CKD-specific component of the KDQOL-36 using the total sample of 506 patients resulted in similar values for the Cronbach's alpha, McDonald's omega, GLB, and Guttman's lambda coefficients, which were between 0.89 and 0.94, indicating a high level of consistency. Likewise, when the analysis was performed for each of the three domains, values for the four coefficients were identified in a range between 0.79 and 0.88. The values obtained for each coefficient in the total sample and according to dialysis modality are shown in Table 5. In the analysis with item removal using the total sample, no increase in Cronbach's alpha or Guttman's lambda coefficients was observed; however, a slight increase in the McDonald’s omega coefficient was observed when item 28 “problems with your access site?” or “problems with your catheter site?” was removed. In the analysis by KRT modality, when item 28 was removed, both in the sample of hemodialysis and peritoneal dialysis patients, a discrete increase in the level of consistency was observed for Guttman’s lambda and McDonald’s omega coefficients. The values of Cronbach's alpha, McDonald's omega, and Guttman's lambda coefficients obtained in the item removal analyses are shown in the Supplementary material, Tables S1, S2, and S3.

**Table 5:** Internal consistency coefficients for the CKD-specific core of the KDQOL-36 scale

	Total sample *n* = 506	Hemodialysis *n* = 253	Peritoneal dialysis *n* = 253
**Cronbach’s Alpha**			
Burden of the kidney disease	0.82	0.82	0.81
Symptoms and problems of kidney disease	0.80	0.81	0.79
Effects of kidney disease	0.82	0.83	0.81
CKD-specific core	0.89	0.89	0.89
**McDonald`s Omega**			
Burden of the kidney disease	0.82	0.82	0.82
Symptoms and problems of kidney disease	0.80	0.81	0.79
Effects of kidney disease	0.82	0.83	0.81
CKD-specific core	0.89	0.89	0.89
**Greatest Lower Bound (GLB)**			
Burden of the kidney disease	0.85	0.86	0.85
Symptoms and problems of kidney disease	0.86	0.88	0.86
Effects of kidney disease	0.86	0.87	0.86
CKD-specific core	0.94	0.95	0.94
**Guttman’s Lambda**			
Burden of the kidney disease	0.79	0.79	0.78
Symptoms and problems of kidney disease	0.81	0.82	0.81
Effects of kidney disease	0.81	0.82	0.81
CKD-specific core	0.91	0.91	0.91

**Fig. 3:**
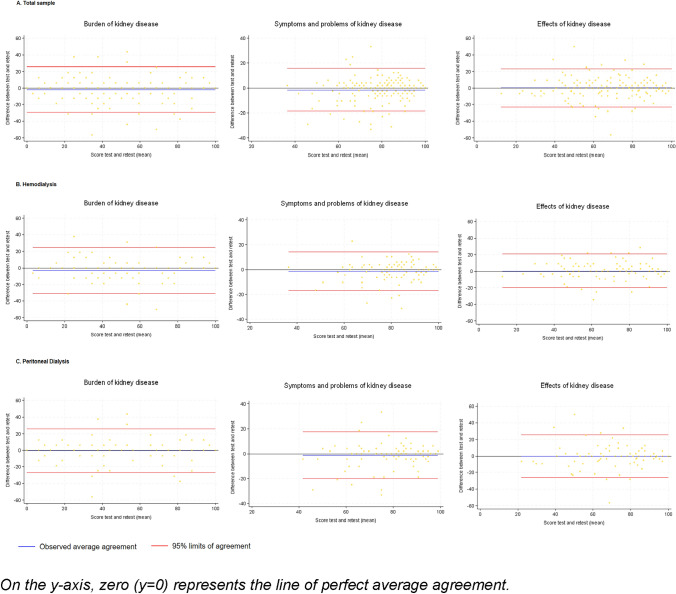
Bland and Altman limits of agreement for the CKD-specific core of the KDQOL-36 scale (On the *y*-axis, zero [*y* = 0] represents the line of perfect average agreement)

***Test–retest reliability***: The KDQOL-36 instrument was applied at a second time to 200 patients, 100 patients on HD, and 100 patients on PD, with a median of 8 days between the two assessments (IQR = 8–10). The analysis of Lin's CCC both in the total sample and by KRT modality for each of the three domains, resulted in all cases in coefficients that were statistically different from zero, with values ≥0.7 (Table 6). In the Bland and Altman plots for each of the three domains, it is evident that the average difference between the first and second application is minimal, with a high level of agreement that remains stable for the entire measurement range of the instrument, being higher for the domain effects of kidney disease (Fig. 3).

**Table 6:** Lin's concordance correlation coefficients for the CKD-specific core of the KDQOL-36 scale

Domain	Lin's CCC*	IC 95%	Bland and Altman limits
**Total sample*n*** **= 200**			
Burden of the kidney disease	0.88	0.84–0.91	−29.049 25.549
Symptoms and problems of kidney disease	0.78	0.73–0.83	−18.516 15.949
Effects of kidney disease	0.82	0.77–0.86	−23.029 23.450
**Hemodialysis*n*** **= 100**			
Burden of the kidney disease	0.87	0.81–0.91	−30.711 24.836
Symptoms and problems of kidney disease	0.80	0.72–0.86	−16.955 14.236
Effects of kidney disease	0.87	0.82–0.91	−20.041 20.899
**Peritoneal Dialysis*n*** **= 100**			
Burden of the kidney disease	0.89	0.85–0.93	−27.315 26.190
Symptoms and problems of kidney disease	0.77	0.69–0.85	−20.027 17.614
Effects of kidney disease	0.77	0.67–0.84	−25.899 25.871

*All Lin's correlation coefficients with *p* values < 0.01

***Sensitivity to change***: The KDQOL-36 instrument was applied at three different times: (I) baseline; (II) when experiencing an event that could modify the quality of life; and (III) once the event was over, again considering the stability of the construct, to 351 patients, 92% in hemodialysis (n = 324 patients) and 8% in peritoneal dialysis (n = 27 patients). The scores obtained in each domain at the three time points are shown in the Supplementary material, Table S4. The analysis using MMRM resulted for each of the three domains in statistically significant differences between the scores obtained with the application of the instrument at different points in time, which shows the instrument's capacity to detect changes in the measurement of the construct as it changes. Coefficients, 95% confidence intervals, and *p* values obtained for each of the three domains are shown in Table 7. Supplementary material, Table S5, shows the values obtained in the pairwise comparisons by dialysis modality.

**Table 7:** Repeated-measure mixed models for the CKD-specific core of the KDQOL-36 scale

	Coefficient^*^	95% CI	p value
**Burden of the kidney disease**			
Time I^a^	Reference		
Time II^b^	2.970	0.300 a 5.641	0.029
Time III^c^	2.874	0.203 a 5.544	0.035
**Symptoms and problems of kidney disease**			
Time I^a^	Reference		
Time II^b^	3.091	1.521 a 4.661	0.000
Time III^c^	3.022	1.452 a 4.592	0.000
**Effects of kidney disease**			
Time I^a^	Reference		
Time II^b^	3.221	0.972 a 5.469	0.005
Time III^c^	5.402	3.153 a 7.651	0.000

*Repeated measures, mixed model adjusted for KRT modality, and the interaction between KRT modality and time of application of the instrument

^a^Baseline

^b^When experiencing an event that could modify quality of life

^c^Once the event is over

## Discussion

Given the need to incorporate QOL as a health outcome during the care of patients with stage 5 CKD on dialysis [78, 79], it is necessary to have evidence of the psychometric properties of the instruments used to measure this construct in each target population [24, 25]. Likewise, it is crucial to generate evidence on the use of more advantageous, complementary, and widely recommended statistical methods to further advance and improve the quality of studies on the measurement properties of PROMs.

The sociodemographic and clinical characteristics of the study population are consistent with the data presented in the latest report published by the Colombian Fund for High-Cost Diseases on the status of CKD in Colombia [5], which additionally reports Bogota as the region with the highest estimated prevalence of KRT in the country, suggesting an adequate representativeness of the study population.

The validity of the instrument was adequate, with evidence of content and concurrent criterion validity. The EFA confirmed the factorial structure proposed in the original instrument for the CKD-specific core of the KDQOL-36, with three factors or domains regarding the burden of kidney disease, physical symptoms, and effects of the disease. For item 28 “problems with your access site?” or “problems with your catheter site?”, no adequate factor loading was found in any of the three factors, with a high uniqueness value; additionally, a discrete increase in the level of consistency was found when it was removed, suggesting that this item could be measuring an aspect other than burden, physical symptoms or effects of the disease, as part of the QoL construct. Despite using a less conservative factor loading threshold than the one used in the present validation, this same finding was evident in the validations carried out in Chinese patients [31], and in Malaysia [35], in which the loading of this item 28 on any of the three identified factors was also not reported. The CFA, performed on an independent subsample, supported the structure of the three factors or domains mentioned, with an adequate fit of the original and exploratory models, finding adequate values for each of the estimators, which were very similar between the two models. Regarding the concurrent criterion validity, despite the differences in the structure and number of items between the CKD-specific core of the KDQOL-36 and the KDQ, an adequate correlation was found between the domains of both instruments, with values of Spearman's correlation coefficient overall ≥0.50. This finding is consistent with what was reported in the validations of the instrument carried out in Arabia, Malaysia, and Ethiopia, which used evidence of different types of validity, such as convergent construct validity and discriminant construct validity [34, 36].

The instrument was reliable, showing evidence of internal consistency and test–retest reliability. In the internal consistency analysis, values for Cronbach's alpha, McDonald's omega, GLB, and Guttman's lambda coefficients were found that indicate a high level of consistency of the three CKD-specific domains of the KDQOL-36. This finding is consistent with what was reported in the studies carried out to validate the instrument in dialysis patients in Thailand [30], China [31], the United States [32], Indonesia [33], Arabia [34], Malaysia [35] and Ethiopia [36] in which Cronbach's alpha coefficient was used as the only measure of internal consistency. It is worth mentioning the discrete increase in the McDonald's omega and Guttman's lambda coefficients when item 28 “problems with your access site?” or “problems with your catheter site?” was removed. Regarding test–retest reliability, the calculation of Lin's concordance correlation coefficient for each of the kidney disease-specific domains of the KDQOL-36, and its corresponding graphical analysis of Bland and Altman limits, allowed us to confirm the stability of the measurements obtained with the instrument at two separate moments in time, considering that the construct remained stable. This finding is consistent with what was reported in validation studies of the instrument carried out in dialysis patients in Thailand [30], China [31], Arabia [34], Malaysia [35], Indonesia [33], and Ethiopia [36] in which evidence of test–retest reliability was generated, although its estimation was carried out using the intraclass correlation coefficient (ICC) or Pearson correlation coefficient. Concerning sensitivity to change, statistically significant differences were found between the scores obtained with the application of the instrument when experiencing an event that could modify the quality of life, which corroborated the instrument's capacity to detect changes in the measurements of the construct as it varies.

The validity and reliability findings of the CKD-specific core of the KDQOL-36 scale in the Colombian population using more advantageous, complementary, and widely recommended statistical methods are consistent with the data presented in validation studies conducted in other countries [30–36]. Additionally, we observed findings of adequate sensitivity to change of the CKD-specific core of the KDQOL-36 scale in the Colombian population, that to the best of our knowledge, at the time of this work, none of the validation studies of the CKD-specific core of the KDQOL-36 scale have included evidence of this psychometric property of the instrument.

A possible limitation of the present study is the small sample size that was possible to obtain for the evaluation of sensitivity to change in PD patients. For further studies of this instrument, we propose the evaluation of sensitivity to change in a larger sample of patients on PD. Also, it is important to evaluate the additional psychometric properties from the perspective of item response theory, such as item- and person-fit indexes, the evaluation of person and item reliability, and the analysis of the coverage of the construct spectrum with the items of the scale.

## Conclusions

The findings from this study allow us to conclude that the KDQOL-36 scale is an instrument with adequate validity, reliability, and sensitivity properties to measure the construct of quality of life in Colombian patients with stage 5 chronic kidney disease on dialysis.

### Supplementary Information

Below is the link to the electronic supplementary material.

Supplementary file1 (DOCX 39 KB)

## Data Availability

The datasets generated and analyzed during the current study are available from the corresponding author on reasonable request.
