# Supplementary material for: Psychometric properties of the Kidney Disease Quality of Life short form 36 (KDQOL-36) scale for the assessment of quality of life in Colombian patients with chronic kidney disease on dialysis
Source: Int Urol Nephrol. 2024 Feb 20;56(7):2337–50. doi: 10.1007/s11255-024-03940-x (PMC11190008; doi:10.1007/s11255-024-03940-x)
Supplement: Supplementary file 1 — Supplementary file1 (DOCX 39 KB) [file 11255_2024_3940_MOESM1_ESM.docx]

**Psychometric properties of the Kidney Disease Quality of Life Short Form 36 (KDQOL-36) scale for the assessment of quality of life in Colombian patients with chronic kidney disease on dialysis.**

**Supplementary material**

**Figure S1.** Box plot with the scores in each of the CKD-specific core domains of the KDQOL-36 scale by KRT modality.

*Each box plot shows the total range from minimum to maximum values, the first and third quartile, and the median value.*

**Table S1.** Cronbach's alpha coefficient for the CKD-specific core of the KDQOL-36 scale with item removal

| **Item removed** | **Total sample** | **Hemodialysis** | **Peritoneal dialysis** |
| --- | --- | --- | --- |
| i13 | 0.89 | 0.88 | 0.88 |
| i14 | 0.89 | 0.89 | 0.88 |
| i15 | 0.89 | 0.89 | 0.88 |
| i16 | 0.89 | 0.89 | 0.88 |
| i17 | 0.89 | 0.89 | 0.89 |
| i18 | 0.89 | 0.89 | 0.89 |
| i19 | 0.89 | 0.89 | 0.89 |
| i20 | 0.89 | 0.89 | 0.89 |
| i21 | 0.89 | 0.89 | 0.89 |
| i22 | 0.89 | 0.89 | 0.89 |
| i23 | 0.89 | 0.89 | 0.89 |
| i24 | 0.89 | 0.89 | 0.88 |
| i25 | 0.88 | 0.88 | 0.88 |
| i26 | 0.89 | 0.89 | 0.89 |
| i27 | 0.89 | 0.89 | 0.89 |
| i28 | 0.89 | 0.89 | 0.89 |
| i29 | 0.89 | 0.89 | 0.89 |
| i30 | 0.89 | 0.89 | 0.89 |
| i31 | 0.88 | 0.88 | 0.88 |
| i32 | 0.89 | 0.88 | 0.89 |
| i33 | 0.89 | 0.89 | 0.89 |
| i34 | 0.88 | 0.88 | 0.88 |
| i35 | 0.89 | 0.89 | 0.89 |
| i36 | 0.88 | 0.88 | 0.88 |

**Table S2.** McDonald’s omega coefficient for the CKD-specific core of the KDQOL-36 scale with item removal

| **Item removed** | **Total sample** | **Hemodialysis** | **Peritoneal dialysis** |
| --- | --- | --- | --- |
| i13 | 0.89 | 0.89 | 0.89 |
| i14 | 0.89 | 0.89 | 0.89 |
| i15 | 0.89 | 0.89 | 0.89 |
| i16 | 0.89 | 0.89 | 0.89 |
| i17 | 0.89 | 0.89 | 0.89 |
| i18 | 0.89 | 0.89 | 0.89 |
| i19 | 0.89 | 0.89 | 0.89 |
| i20 | 0.89 | 0.89 | 0.89 |
| i21 | 0.89 | 0.89 | 0.89 |
| i22 | 0.89 | 0.89 | 0.89 |
| i23 | 0.89 | 0.89 | 0.89 |
| i24 | 0.89 | 0.89 | 0.89 |
| i25 | 0.88 | 0.88 | 0.88 |
| i26 | 0.89 | 0.89 | 0.89 |
| i27 | 0.89 | 0.89 | 0.89 |
| i28 | **0.90** | **0.90** | **0.90** |
| i29 | 0.89 | 0.89 | 0.89 |
| i30 | 0.89 | 0.89 | 0.89 |
| i31 | 0.89 | 0.89 | 0.89 |
| i32 | 0.89 | 0.89 | 0.89 |
| i33 | 0.89 | 0.89 | 0.89 |
| i34 | 0.88 | 0.88 | 0.88 |
| i35 | 0.89 | 0.89 | 0.89 |
| i36 | 0.89 | 0.89 | 0.89 |

**Table S3.** Guttman's lambda coefficient for the CKD-specific core of the KDQOL-36 scale with item removal

| **Item removed** | **Total sample** | **Hemodialysis** | **Peritoneal dialysis** |
| --- | --- | --- | --- |
| i13 | 0.90 | 0.91 | 0.91 |
| i14 | 0.90 | 0.91 | 0.91 |
| i15 | 0.90 | 0.91 | 0.91 |
| i16 | 0.90 | 0.91 | 0.91 |
| i17 | 0.90 | 0.91 | 0.91 |
| i18 | 0.91 | 0.91 | 0.91 |
| i19 | 0.91 | 0.91 | 0.91 |
| i20 | 0.90 | 0.91 | 0.91 |
| i21 | 0.90 | 0.91 | 0.91 |
| i22 | 0.90 | 0.91 | 0.91 |
| i23 | 0.91 | 0.91 | 0.91 |
| i24 | 0.90 | 0.91 | 0.91 |
| i25 | 0.90 | 0.90 | 0.90 |
| i26 | 0.91 | 0.91 | 0.91 |
| i27 | 0.90 | 0.91 | 0.91 |
| i28 | 0.91 | **0.92** | **0.92** |
| i29 | 0.90 | 0.91 | 0.91 |
| i30 | 0.90 | 0.91 | 0.91 |
| i31 | 0.90 | 0.91 | 0.91 |
| i32 | 0.90 | 0.91 | 0.91 |
| i33 | 0.90 | 0.91 | 0.91 |
| i34 | 0.90 | 0.91 | 0.90 |
| i35 | 0.91 | 0.91 | 0.91 |
| i36 | 0.90 | 0.91 | 0.91 |

**Table S4.** Scores at each of the three moments of application for each of the domains of the CKD-specific core of the KDQOL-36 scale by KRT modality

|  | **Time I ^1^** | **Time II ^2^** | **Time III ^3^** |
| --- | --- | --- | --- |
| **Total sample n = 351** | | | |
| **Burden of the kidney disease** | 43.75 (18.75 – 68.75) | 43.75 (18.75 – 75) | 43.75 (25 – 75) |
| **Symptoms and problems of kidney disease** | 81.25 (66.67 - 91.67) | 83.33 (72.92- 91.67) | 83.33 (70.83 – 91.67) |
| **Effects of kidney disease** | 65.63 (50 – 84.38) | 71.88 (50 – 87.5) | 71.88 (56.25 – 87.5) |
| **Hemodialysis n = 324** | | | |
| **Burden of the kidney disease** | 43.75 (18.75 – 65.62) | 43.75 (21.87 – 75) | 43.75 (25 – 75) |
| **Symptoms and problems of kidney disease** | 81.25 (66.67- 91.67) | 84.37 (72.92- 93.75) | 83.33 (70.83 – 91.67) |
| **Effects of kidney disease** | 65.63 (50 – 84.38) | 71.88 (53.13 – 87.5) | 73.44 (59.38 – 90.63) |
| **Peritoneal Dialysis n = 27** | | | |
| **Burden of the kidney disease** | 50 (25 – 75) | 34.37 (12.5 – 68.75) | 50 (25 – 62.5) |
| **Symptoms and problems of kidney disease** | 83.33 (64.58 – 89.58) | 70.83 (54.16- 81.25) | 85.41 (72.91 – 93.75) |
| **Effects of kidney disease** | 65.62 (46.87 – 81.25) | 56.25 (31.25 – 78.12) | 59.37 (50 – 78.12) |

*All data are presented as median (IQR). 1. Baseline. 2. When experiencing an event that could modify quality of life. 3. Once the event is over.*

**Table S5.** Repeated-measure mixed models for the CKD-specific core of the KDQOL-36 scale, pairwise comparisons by KRT modality

|  | **Coefficient ^*^** | **95 % CI** | ***p* value** |
| --- | --- | --- | --- |
| **Hemodialysis, n = 320** | | | |
| **Burden of the kidney disease** | | | |
| Time I ^1^ vs Time II^2^ | 2,970 | 0,300 a 5,641 | 0,029 |
| Time III ^3^ vs Time II^2^ | - 0,096 | - 2,766 a 2,574 | 0,944 |
| **Symptoms and problems of kidney disease** | | | |
| Time I ^1^ vs Time II^2^ | 3,091 | 1,521 a 4,661 | 0,000 |
| Time III ^3^ vs Time II^2^ | - 0,069 | - 1,639 a 1,501 | 0,931 |
| **Effects of kidney disease** | | | |
| Time I ^1^ vs Time II^2^ | 3,221 | 0,972 a 5,469 | 0,005 |
| Time III ^3^ vs Time II^2^ | 2,181 | -0,067 a 4,430 | 0,057 |
| **Peritoneal Dialysis, n = 27** | | | |
| **Burden of the kidney disease** | | | |
| Time I ^1^ vs Time II^2^ | - 9,139 | - 18,513 a 0,235 | 0,056 |
| Time III ^3^ vs Time II^2^ | 9,139 | - 0,235 a 18,513 | 0,056 |
| **Symptoms and problems of kidney disease** | | | |
| Time I ^1^ vs Time II^2^ | - 8,228 | - 13,667 a - 2,789 | 0,003 |
| Time III ^3^ vs Time II^2^ | 12,885 | 7,446 a 18,324 | 0,000 |
| **Effects of kidney disease** | | | |
| Time I ^1^ vs Time II^2^ | - 6,828 | - 14,618 a 0,961 | 0,086 |
| Time III ^3^ vs Time II^2^ | 6,828 | - 0,961 a 14,618 | 0,086 |

** Repeated measures, mixed model adjusted for KRT modality, and the interaction between KRT modality and time of application of the instrument.* *1. Baseline. 2. When experiencing an event that could modify quality of life. 3. Once the event is over.*
